# Analysis of acid–base disorders in an ICU cohort using a computer script

**DOI:** 10.1186/s40635-022-00437-8

**Published:** 2022-04-04

**Authors:** Innas Forsal, Mikael Bodelsson, Anders Wieslander, Anders Nilsson, Dominique Pouchoulin, Marcus Broman

**Affiliations:** 1 Baxter Gambro Lundia, Magistratsvägen 16, 22643 Lund, Sweden; 2 Skåne University Hospital Perioperative and Intensive Care, Entregatan 7, 22242 Lund, Sweden; 3 Baxter France, 7 Av. Lionel, 69330 Meyzieu, France

**Keywords:** Acid–base disturbance, Acidosis, Alkalosis, Homeostasis, Acute kidney injury, Critical care

## Abstract

**Background/aims:**

Acid–base status is important for understanding pathophysiology, making a diagnosis, planning effective treatment and monitoring progress of critically ill patients. Manual calculations are cumbersome, easily result in wrong conclusions. We wanted to develop an automated assessment of acid–base status.

**Methods:**

A simplified adaptive MATLAB script processing all available theory to date was created, evaluated and used on blood gas analyses drawn immediately after admission to ICU. The script was compared to golden standard, calculating manually by two experienced ICU physicians.

**Results:**

Results from the script correlated completely with detailed manual calculations of randomly chosen 100 blood gas results and it was able to deliver complex data on cohort level with advanced graphics. The initial blood gas analyses from 8875 admissions constituted the cohort, of which 4111 (46.3%) were normal. Respiratory acidosis was the primary disturbance in 2753 (31.0%) and metabolic acidosis in 464 (5.2%). Respiratory alkalosis was the primary disturbance in 1501 (17.0%) and metabolic alkalosis in 46 (0.5%). Of the disturbances 74.7% were mixed with two and 2.1% with three simultaneous disturbances. Acidoses were less compensated compared to alkaloses.

**Conclusions:**

Acid–base theories are developed on ideal models and not on critical care patients, they require inputs that might not be available, and therefore, estimations are needed. In our cohort, it was difficult to develop a working script based on Stewart, whereas Boston/Copenhagen worked better. Acidoses were more common and more deviated compared to alkaloses.

## Introduction

Accurate interpretation of acid–base status is of great importance for understanding pathophysiology, making a diagnosis, planning effective treatment and monitoring progress of a critically ill patient. To do calculations manually is cumbersome and results in wrong conclusions. Often several blood gas analysis results are needed to understand dynamic processes. Attempts to simplify the acid–base diagnosis have been developed, but still a general method with a good clinically instant and understandable result is lacking. Most acid–base disturbances are mild and easily overseen by the clinicians [1, 2] but can be the first sign of a detrimental process starting.

No data of the spectrum of acid–base changes occurring in a large cohort consisting of critically ill patients is available, simply because the large number of calculations cannot be carried out manually.

We have combined physiological understanding with mathematical processing to create an overall simplified script providing both numerical and graphic output, using a modern mathematical software (MATLAB®2020a).

In addition, the script should be written in a format that facilitates it to be used in an application for a handheld phone or a computer, as well as to be incorporated in the software of any device used in health care.

The script should be able to perform a single calculation with a clinical meaningful output as well as being able to handle several thousand blood gases and deliver its output in a database format. In this way the script can serve bedside in the clinic as well as a tool to analyse large cohorts for research purposes.

The aims of this study were to establish descriptive data on the initial blood gas upon arrival to the ICU and to analyze appearance of acid–base disturbances on a very large ICU cohort, using and testing the script that we have developed.

## Methods

### Acid–base theory

Acidity of aqueous solutions is determined by measuring the hydrogen ion (H^+^) activity, which is related to its concentration. The hydroxide ion (OH^−^) is the counterpart to H^+^ and increased concentration equals increased alkalinity. A solution is neutral when [H^+^]=[OH^−^], with a negative logarithm of the H^+^ concentration of 7.0, the pH value, which is used to describe the concentration.

Blood in normal acid–base balance has a pH of 7.4 and is alkalotic with [H^+^]<[OH^−^]. In a medical context, states with higher H^+^ concentrations than this are defined as acidosis, whereas states with lower H^+^ concentrations are defined as alkalosis.

Hydrogen ion concentration in blood is tightly regulated by a system of very complex mechanisms to a pH value of 7.4, consisting of basically three players; (1) buffer molecules which react with hydrogen ions deactivating them and (2) the respiration by increasing or lowering the pCO_2_ with hypo- or hyperventilation and (3) the renal function by excretion or retention of bicarbonate. These compensation mechanisms come into play immediately, but act with different speed, renal is the slowest. The aim is a pH of 7.4, and overcompensation can never occur [3–9].

### The Boston and Copenhagen approach

In the Boston approach bicarbonate is the most important buffer with its ability to bind or release hydrogen ions to keep the pH as constant as possible. The Henderson–Hasselbach equation provides a simple relationship between carbon dioxide (the respiratory component), bicarbonate (the non-respiratory component) and pH. Its weakness is that it takes into account bicarbonate as the only buffer, and neglects the impact of other buffers, such as albumin and hemoglobin [3, 7, 10, 11].

Base excess was introduced in the Copenhagen approach by Siggaard–Andersen, as a parameter separating the metabolic from the respiratory component. It is defined as the amount of acid or alkali to be added to achieve pH 7.40, given that the pCO_2_ is constant. Base excess in blood, which also takes into account the buffering effect of hemoglobin, gives the level of the metabolic component independently from the respiratory component [8, 9, 12].

### Stewart approach

The Stewart method calculates on a system consisting of (1) strong ions with individual constant charges over a physiological range; measured by the strong ion difference (SID), (2) non-volatile proton donor/acceptors, which transfer hydrogen ions within a physiological pH range, i.e., weak acids/bases; defined by the concentration of weak acids (A_TOT_) and (3) the volatile bicarbonate–CO_2_ buffer system; defined by the partial pressure of carbon dioxide (pCO_2_).

A falling SID correlates with increased acidemia, whereas a rising SID correlates with increased alkalemia, Blood in normal state is alkalotic with pH 7.40 and SID 42 mEq/L. A_TOT_ represents all non-bicarbonate buffers and an increase results in metabolic acidosis, whereas a decrease leads to metabolic alkalosis. The respiratory component is represented by the carbon dioxide partial pressure, an increase leads to acidosis and a decrease leads to alkalosis [13, 14].

### Our own simplified combined numerical and graphic approach

We argue that all theories described above; Boston, Copenhagen and Stewart are not easy to fully understand and use in everyday interpretation of patients’ acid–base status, and clinicians must rely on formulas that are often rough simplifications leading to wrong interpretations in especially complex acid–base disturbances. Therefore, we developed a computer script, which uses all available theory and that can interpret an individual blood gas in full, and deliver a detailed linguistic, numerical and graphic output. The script can also handle an infinite number of blood gases and deliver the output to a database.

The script has a basic structure of four levels of complex calculation steps and the output from each level is transferred to the next; however, the final level accepts input from all previous levels. The script estimates a most probable start-scenario for a certain pH/pCO_2_ combination, on which further calculations are carried out in subsequent levels, as shown in Tables 1 and 2. The layers 1–3 are straightforward and follow basic acid–base theory. The fourth and last layer does pattern recognition tasks, compares values to each other and is possible to extend for future versions of the script with machine learning and adaptive loops.

**Table 1:** Conditional flow sheet for determination of the most probable primary disturbance in initial level one of the algorithm

pCO_2_	pH	Outcome
⇩	⇩	Metabolic acidosis
⇩	⇧	Respiratory alkalosis
⇧	⇩	Respiratory acidosis
⇧	⇧	Metabolic alkalosis

**Table 2:** Description of the structure of the algorithm

Level	Function
1 ⇩	Initial input variables are pCO_2_ and pH. The script estimates a most probable start-scenario including a primary disturbance for certain pH/pCO_2_ combinations (Table 1), on which further calculations are carried out in the next level
2 ⇩	The input variables are the primary disturbances from level 1. The compensation variables; pCO_2_ for metabolic and bicarbonate for respiratory disturbances are determined. Full compensation (100%) is defined according to the Boston formulas. If the compensation is partial the script defines a second disturbance that interacts with/counteracts the compensation mechanism
3 ⇩	The input variable is the diagnosis of metabolic acidosis from level 2. The script calculates the anion gap and the delta ratio to evaluate a possible tertiary disturbance in case of a primary or secondary disturbance of metabolic acidosis. Anion gap is calculated as [Na^+^] + [K^+^] − [Cl^−^] − [bicarbonate]. The traditional delta ratio was further calculated for all anion gaps > 16 using the formula [AG-12]/[24-bicarbonate]
⇩⇩⇩ 4	The results from the previous levels 1–2–3 are inserted in a final fourth evaluation filter, where the diagnosis is compared to special criteria. To date the fourth level is not fully built and it serves as possibility to further develop the script. Finally, a graph is drawn, where pH is placed on the *X* axis giving an instant understanding whether the result is an acidosis or an alkalosis. Bicarbonate level is placed on the *Y* axis. In addition, a superficial layer consisting of pCO_2_ isopleths, created using the Henderson–Hasselbach equation solved for bicarbonate, are placed on top of the graph

After permission was obtained from The Swedish Ethical Review Authority (Dnr 2020-04642) 8875 initial blood gas analyses during years 2011–2021 at Skåne University Hospital, Adult Critical Care Unit, drawn immediately after the admission to ICU, were extracted from the medical records ICCA system (Philips, Vienna, Austria) using an extraction client, and included in the study cohort on which the script was applied.

### Statistics

Two sample *t* test was used by carrying out the function *t* test2 on MATLAB. Significance level was decided to *p*-value < 0.01.

### Validation of model

One hundred randomly chosen blood gas results were calculated and determined in depth by two experienced intensivists, and then the script calculated the same blood gases.

## Results

### Output of the script on individual level

**Fig. 1:**
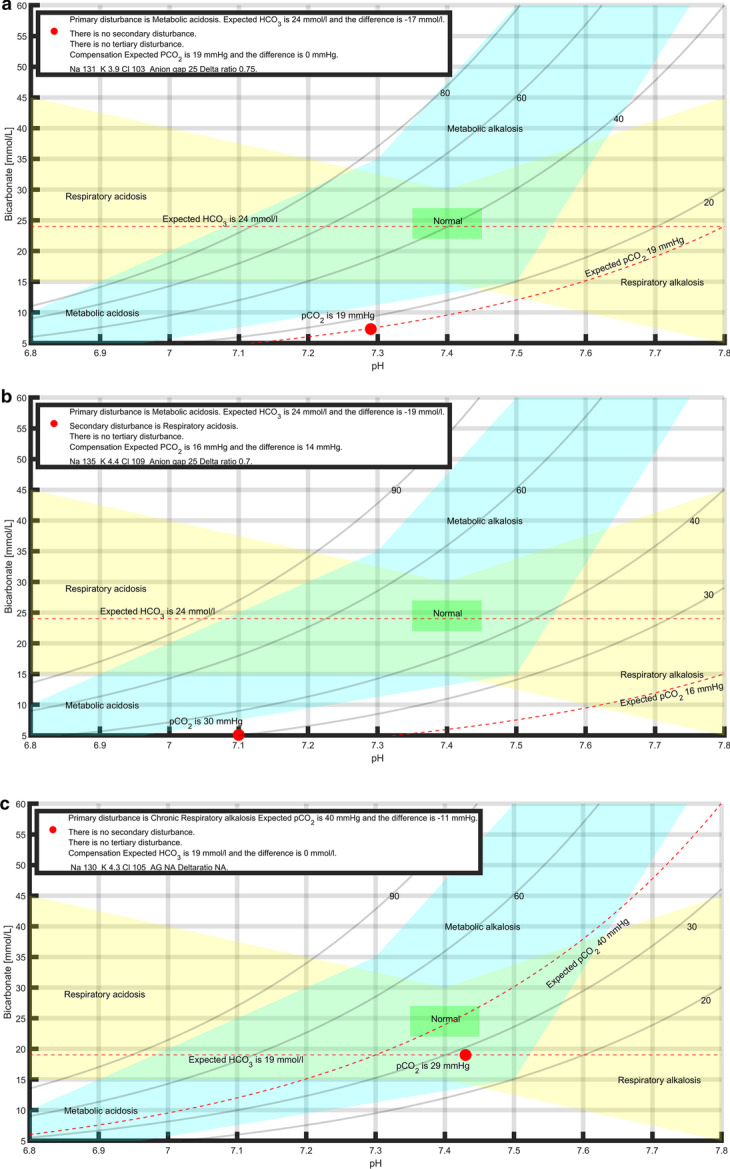

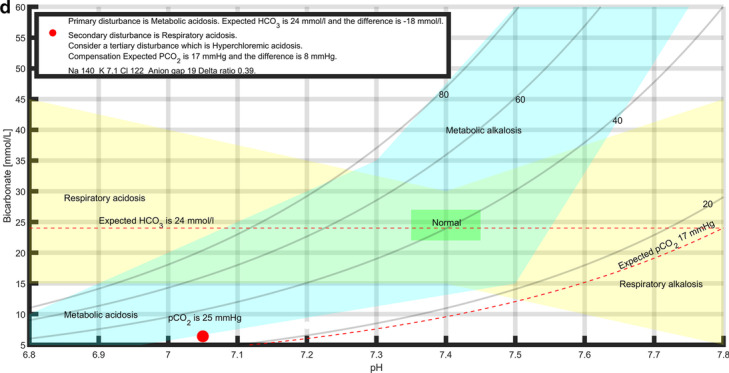
**A** Primary disturbance is a metabolic acidosis and the deviation from baseline is − 17 mmol/L. The compensation is full with a hyperventilation resulting in a pCO_2_ of 19 mmHg. The script uses Boston formulas. The graphic output shows the low bicarbonate and the low pCO_2_ levels, as well as the pH which has been compensated close to normal. **B** Primary disturbance is a metabolic acidosis and the deviation from baseline is − 19 mmol/L, which equals a critical metabolic acidosis. However, in this case, there is a secondary respiratory acidosis disturbance making it impossible for the patient to hyperventilate the pCO_2_ down to 16 mmHg, which would be a complete compensation. Instead, the pCO_2_ has remained at 30 mmHg level. This means that the compensation is 10 mmHg (equals actual compensation = [40–30] mmHg against full compensation = [40–16] mmHg and thus 42 percent). **C** Primary disturbance is a chronic respiratory alkalosis and the deviation from baseline is − 11 mmHg. Because the compensation overshoots the level of full compensation for acute respiratory alkalosis, which would equal a bicarbonate level of 22 mmol/L (instead it is 19 mmol/L) the disturbance is chronic. In fact this equals full compensation for chronic disturbance and the script thus returns that the primary respiratory alkalosis disturbance is chronic, fully compensated and that no secondary disturbance is identified. Also, the pH is within normal range. **D** Primary disturbance is a metabolic acidosis with a deviation from the baseline of − 18 mmol/L, with an elevated anion gap of 19. The script returns a secondary disturbance of a respiratory acidosis, because the compensation is not full; there is a 8 mmHg difference (actual pCO_2_ value is 25 mmHg) to full which would be 17 mmHg. In this case the delta ratio is calculated to 0.39, which indicates a tertiary disturbance. The [Na^+^–Cl^−^] difference is 18 mmol/L and the script returns a suggestion of a tertiary disturbance as a hyperchloremic acidosis

The output of the script is a simplistic integration consisting of a numerical, linguistic and graphic presentation. The numerical output is interpreted in words. Primary, secondary and tertiary disturbances as well as the level of primary disturbance and the difference to full compensation are presented. A graph is drawn, where pH is on the *X* axis giving an instant understanding whether the result is an acidosis or an alkalosis. Bicarbonate level is on the *Y* axis. In addition, a superficial layer consisting of pCO_2_ isopleths, created using the Henderson–Hasselbach equation solved for bicarbonate, are drawn defining the isopleth of the actual value of the blood gas, which is used as the reference for the pCO_2_ scale [15, 16]. The blood gas itself is represented by a red dot. The normal level, from which the primary disturbance has deviated, is drawn as a dotted line. The expected level for full compensation is also drawn as a dotted line. A visual understanding how far from a green normal area, and how far from full compensation, is shown for the blood gas. Four examples of outputs are presented in Fig. 1A–D.

One hundred blood gas results were calculated and determined in depth by two experienced intensivists, and then the script calculated the same blood gases. The diagnoses were completely in accordance. The script calculated a single blood gas instantly, compared to manual calculating which required 1–10 min for a simple and a complicated disturbance, respectively. The time frame for the script to calculate all 8875 blood gases in the cohort was 30.3 s. The script contains ~ 500 rows, there is 1 calculation per row, giving 8875 × 500 = 4,437,500 calculations for the complete cohort. To do the same calculation maneuver manually is impossible.

### Output of the script on a cohort level

Of the 8875 blood gases analyzed 4111 (46.3%) were considered normal. Respiratory acidosis was the primary disturbance in 2753 (31.0%) and metabolic acidosis in 464 (5.2%). Respiratory alkalosis was the primary disturbance in 1501 (17.0%) and metabolic alkalosis in 46 (0.5%).

4764 (53.7%) blood gases showed an acid–base disturbance. Of these a majority presented a mixed disturbance; 3558/4764 (74.7%) patients had a primary + secondary disturbance and 100/4764 (2.1%) patients had a mixed situation with a primary + secondary + tertiary disturbance.

### Level of deviation from normal and level of compensation

Respiratory acidoses (*p* < 0.01) and metabolic acidoses (*p* < 0.01) are more deviated from normal, compared to the corresponding alkaloses, as shown in Table 3.

**Table 3:** Levels of deviation for the different disturbances

Deviation	Normal value	Value [mean ± SD, most extreme]	Value [mean ± SD, most extreme]
Respiratory acidosis *n* = 2753	pCO_2_ 40 mmHg pH 7.40	60.2 ± 17.2 mmHg Highest 206 mmHg	pH 7.22 ± 0.099 Lowest 6.49
Respiratory alkalosis *n* = 1501	pCO_2_ 40 mmHg pH 7.40	29.8 ± 3.6 mmHg Lowest 14 mmHg	pH 7.50 ± 0.033 Highest 7.64
Metabolic acidosis *n* = 464	Bicarbonate 24 mmol/L pH 7.40	14.5 ± 6.9 mmol/L Lowest 5.4 mmol/L	pH 7.20 ± 0.133 Lowest 6.73
Metabolic alkalosis *n* = 46	Bicarbonate 24 mmol/L pH 7.40	43.0 ± 2.9 mmol/L Highest 47.4 mmol/L	pH 7.49 ± 0.035 Highest 7.59

**Fig. 2:**
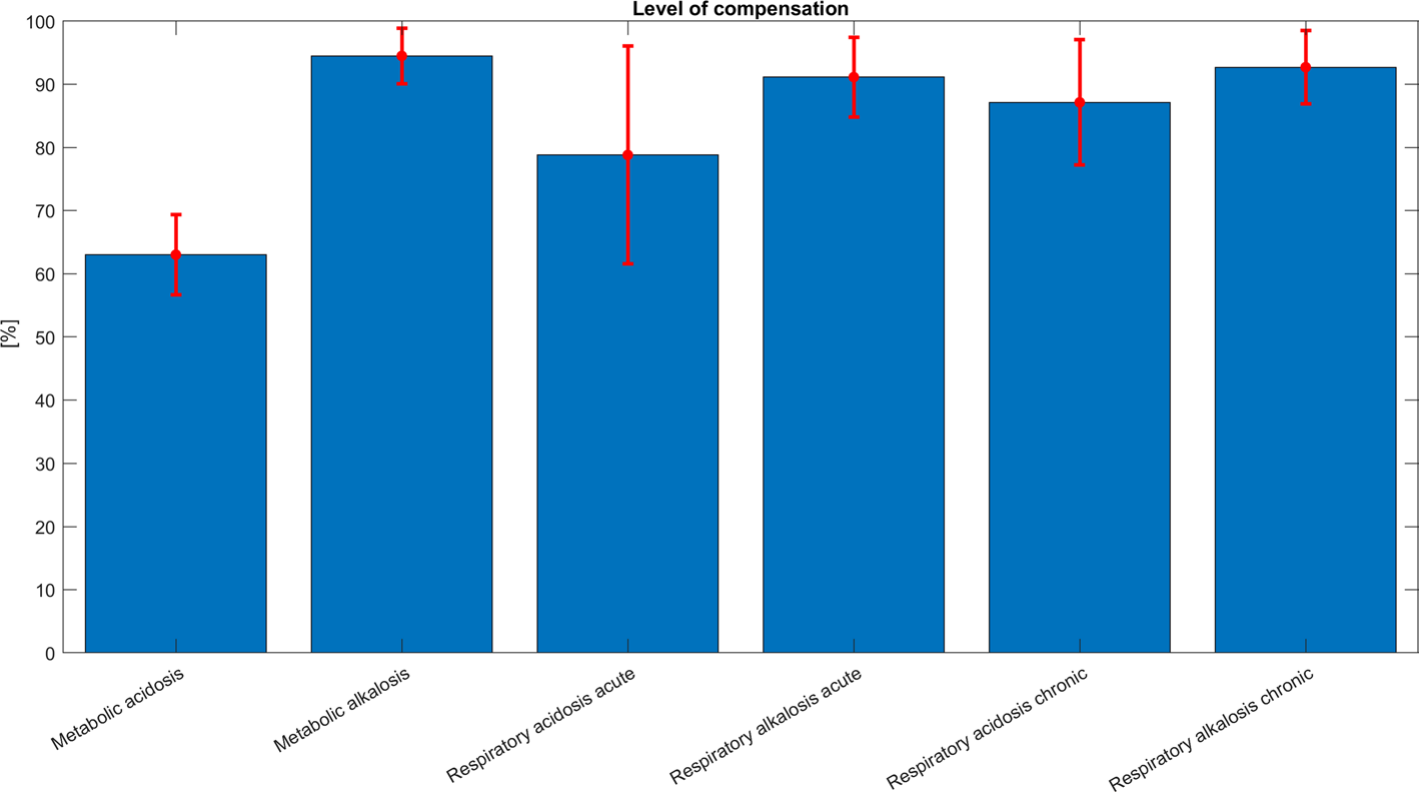
Metabolic, acute and chronic respiratory acidoses were less compensated compared to corresponding alkaloses (*p* < 0.01). The percentage of the compensation ± SD is shown on the *Y* axis. Full compensation (100%) is defined according to Boston rules, and the actual compensation is defined as a percentage of full compensation

Acidoses were significantly less compensated compared to alkaloses, both in respiratory (*p* < 0.01) and metabolic (*p* < 0.01) disturbances, as shown in Fig. 2. Deviation was calculated by taking into account the shift in pCO_2_ from 40 mmHg or bicarbonate shift from 24 mmol/L, as well as the total pH shift from 7.4. Compensation was calculated using the Boston formulas [17].

### Tertiary disturbance

**Fig. 3:**
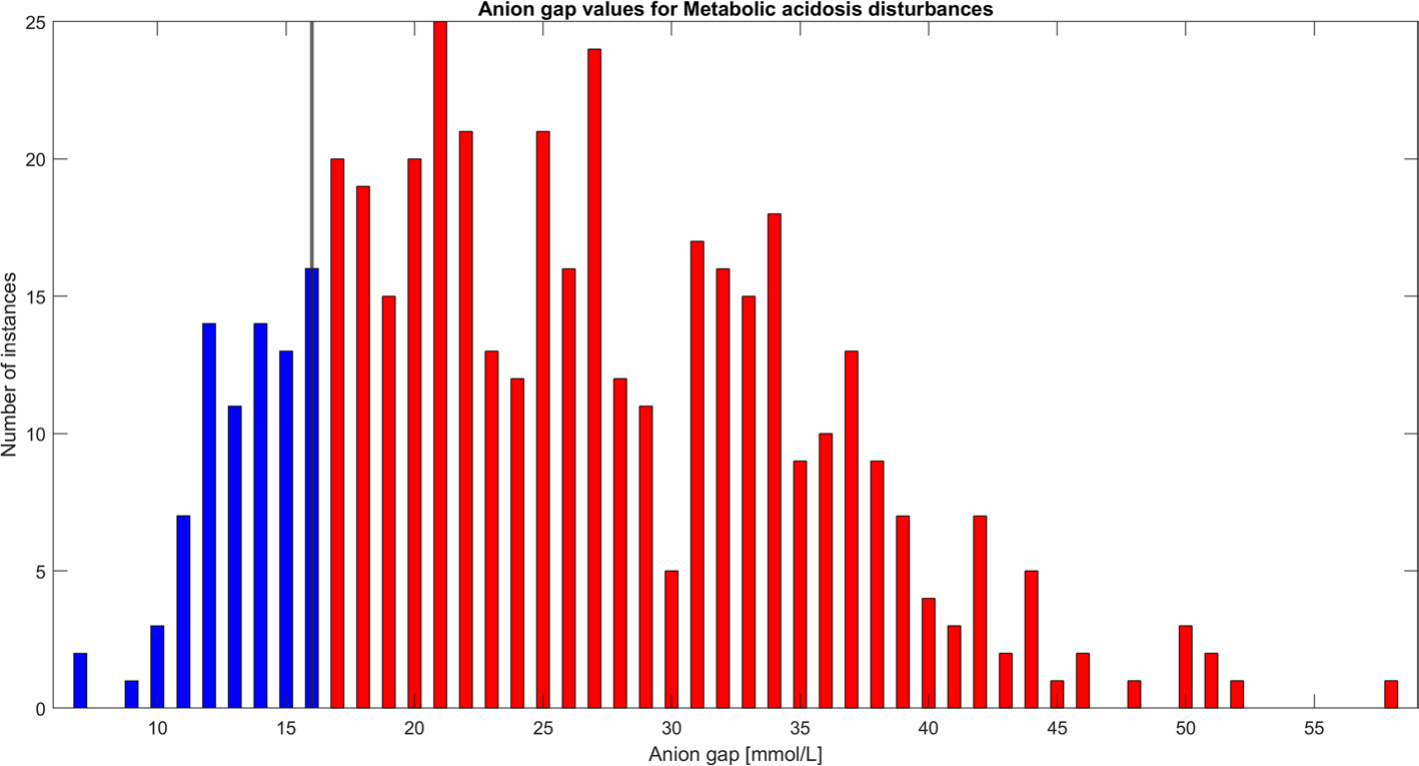
Eighty-three percent of the metabolic acidoses showed an elevated anion gap > 16 mmol/L. The distribution of the anion gaps is shown; < 16 in blue and > 16 in red

Most part of the metabolic acidoses (385/464, 83.0%) showed an elevated anion gap, calculated as [Na^+^] + [K^+^] − [Cl^−^] − [bicarbonate]. The distribution of the anion gaps is shown in Fig. 3.

**Fig. 4:**
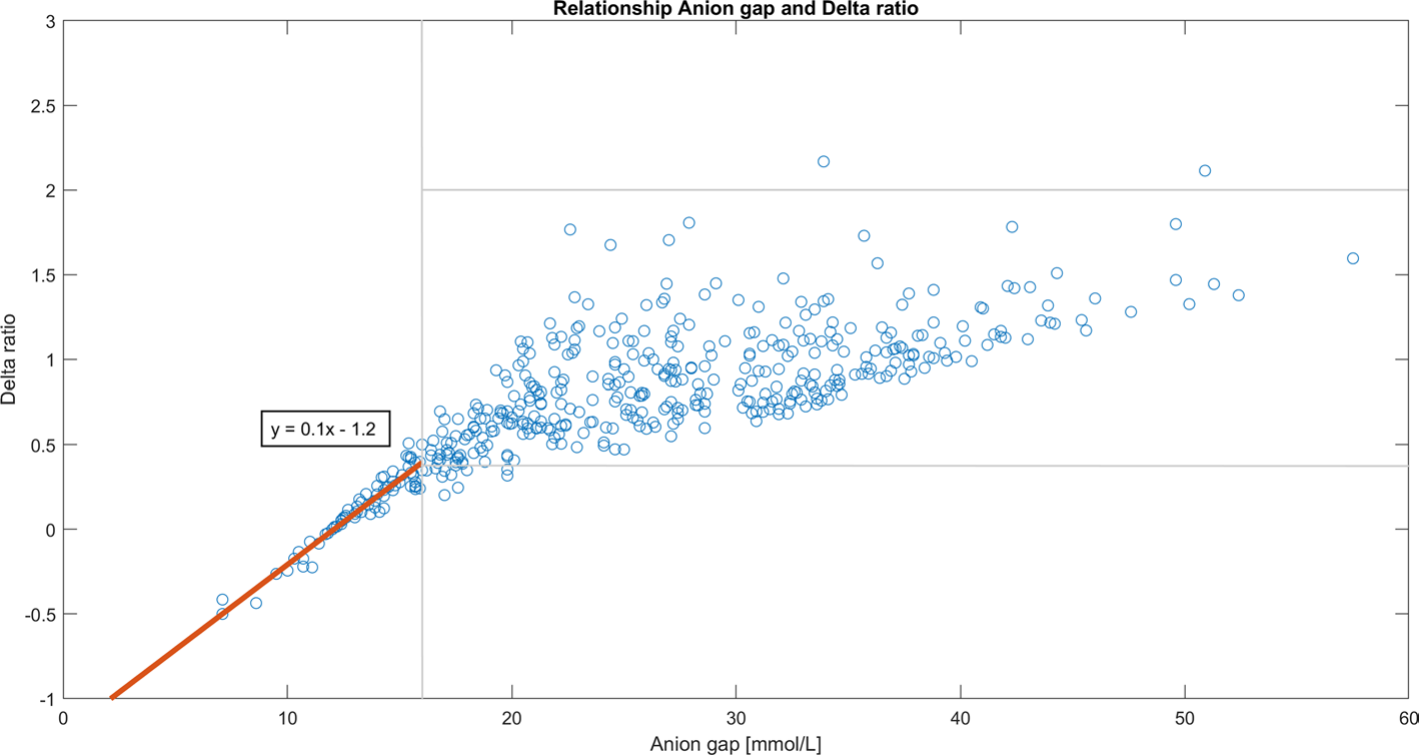
For anion gaps above 16 the delta ratios deviated making it possible to divide them into different ranges indicating a disturbance in the bicarbonate compensation of the elevated anion gap

The traditional delta ratio was calculated for all anion gaps using the formula [AG-12]/[24-bicarbonate] [9, 10]. The result is shown as a plot in Fig. 4. For normal anion gaps up to 16 there is a tight linear relationship between the delta ratio described by the equation *y* = 0.1*x* + 1.2, where *y* = delta ratio and *x* = anion gap. For elevated anion gaps the delta ratios sprawl between 0 and 2. A normal anion gap is built up from phosphate and albumin mainly and compensated by stoichiometric alteration of the bicarbonate. According to existing theory delta ratios < 0.4 indicate a tertiary hyperchloremic acidosis and delta > 2 indicate a pre-existing high bicarbonate state, i.e., a metabolic alkalosis or a compensation state on a respiratory acidosis. Our data favors that the delta ratios can point out and define a third add-on disturbance. The delta ratio is to be considered as an approximation and should always be interpreted with great care [13, 14].

### Strong ion difference

Strong ion difference (SID) was estimated (SID_e_) using available parameters in our raw material [Na^+^] + [K^+^] − [Cl^−^] − [lactate^−^] [13, 14]. According to Stewart theory at any given pCO_2_, a falling SID or a rising A_TOT_ will reduce pH and cause acidosis. In our raw material by estimating strong ion difference we discovered a usable relationship neither in acidotic, nor in alkalotic blood gases.

**Fig. 5:**
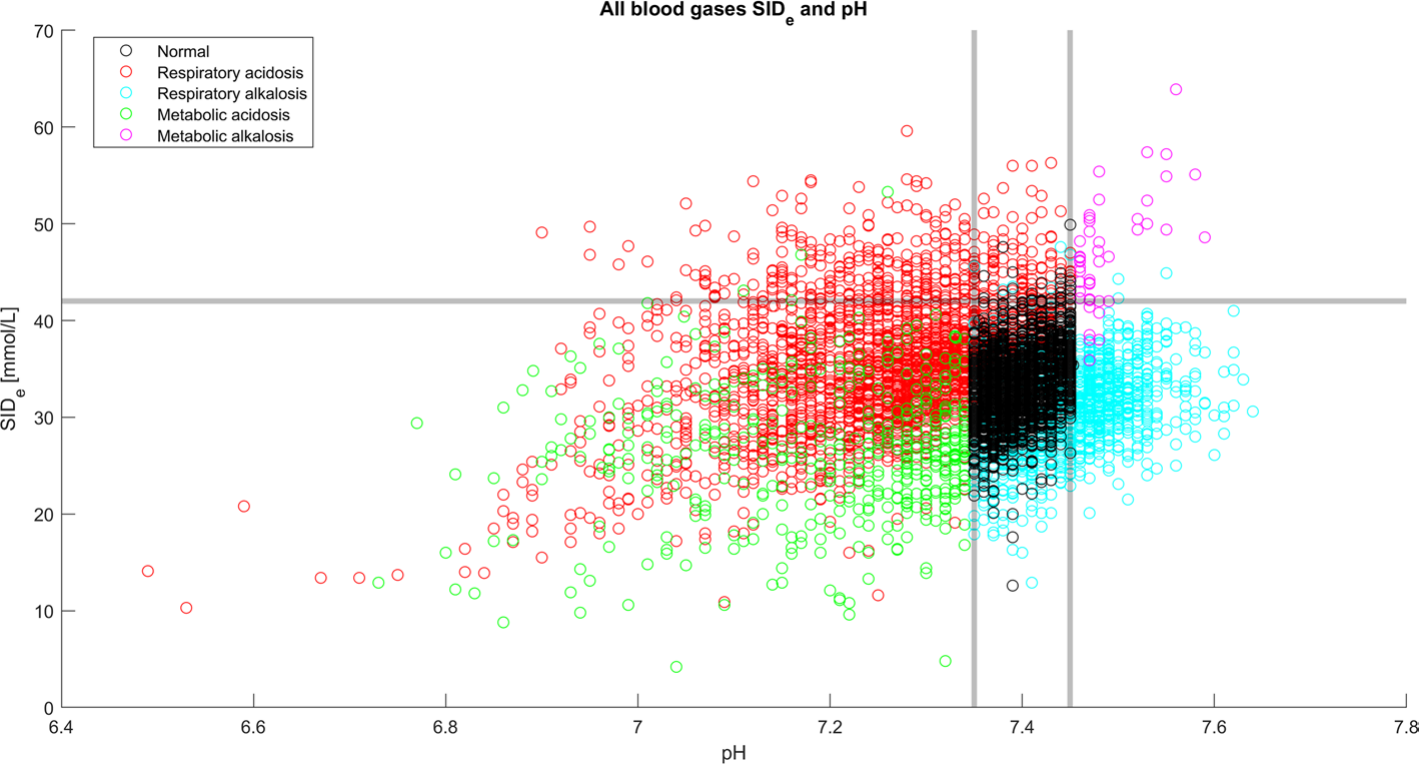
SID_e_ (estimated) did not correlate with pH in the cohort. SID_e_ did not either correlate with pH for exclusively metabolic acidoses (green) nor for metabolic alkaloses (purple). Blood gases with normal pH are shown in black

A falling SID_e_ should ideally indicate an increasing level of metabolic acidosis. Our SID-estimate did not match the acid–base status as shown in Fig. 5, with a *r* square of 0.04 for all blood gases in the cohort, 0.01 for metabolic acidoses and 0.39 for metabolic alkaloses for a significance level of < 0.01. In blood gases with normal pH 7.35–7.45 mean SID_e_ was 33.1 ± 5.5 mmol/L (median 33.1).

## Discussion

We created a mathematical script that can interpret blood gas results on an individual as well as on a cohort level, using all the available theory, and deliver a clinically meaningful result. Basically, any parameter or relation can be calculated.

We argue that a physician in a clinical situation does rarely have time to manually calculate complex acid–base disturbances, and the risk is considerable that the result is not correct.

A script allows for a precise and fast interpretation on a single blood gas around the clock, and it can also analyse blood gas results' correlation to underlying diseases and to monitor ongoing critical care on cohort level. There is basically no limit how large cohorts the script can handle but the device's capacity can constitute a limitation, which especially applies to a handheld device.

To achieve as a homogeneous cohort as possible we inserted the first blood gas taken after admission to the ICU into the script. A total of 8875 blood gases were included.

The script was evaluated by comparing 100 randomly chosen blood gases calculated and determined by two experienced intensivists, to the output of the script, and the diagnoses were the same in all instances. The script was stable and could easily calculate 8875 bloodgases. In fact it was tested successfully on 300 k bloodgases.

The script first inserted the pCO_2_ level and corresponding pH in a conditional flow-sheet system. The output was matched against the bicarbonate level. This output constituted the primary disturbance.

Then the compensation process was defined as the opposite to the primary disturbance. If the level of compensation does not reach full and remains as partial, there must be a second disturbance interacting with the compensation. This version of the script uses traditional Boston formulas to determine whether the compensation is full or partial [17]. Acute versus chronic is eligible for respiratory disturbances only. In real life a timescale is used for differing acute and chronic disturbances, less than 3 days acute otherwise chronic. The renal buffering, by altering the bicarbonate level to counteract the impact on pH, is in respiratory acidosis 4 times higher in a chronic compared to an acute setting, and in respiratory alkalosis 5/2 times lower in a chronic setting, to reach full compensation [10]. The acute/chronic concept is not eligible in metabolic disturbances, because there is a respiratory compensation mechanism which is always instant.

A tertiary disturbance is diagnosed via the delta ratio. It was used for diagnostics only if the anion gap was > 16. For normal anion gaps the relation to the delta ratio is linear, while it for elevated anion gaps diverged ~ between 0 and 2.2, and thus can indicate that an interaction must have taken place if the delta ratio is less than 0.4 or higher than 2. The latter occurred only two times in this cohort, raising the question if 2 is the correct cutoff. Existing theory is based on the fact that bicarbonate is the only buffer counteracting the anion gap and that no intra-cellular buffering takes place. This is not true, because hemoglobin, as well as other proteins to a lesser extent, buffer also, and intracellular buffering actually takes place [1, 13, 15, 16]. Therefore, delta ratio is to be considered as indicative. In the first version of the script we inserted the criteria; (1) if delta ratio is < 0.4 AND [Na^+^ − Cl^−^] < 40 mmol/L a diagnosis of hyperchloremic acidosis is decided, and (2) if delta ratio > 2 a diagnosis of a pre-existing high-level bicarbonate state is decided. Such high-level bicarbonate state can originate from a true pre-existing high level or from a longtime compensation of a chronic respiratory insufficiency.

The fourth and last layer compares values to each other and is possible to extend for future versions of the script with machine learning. It can accept the diagnosis from the 1 to 3 levels, or alter key values and require a new calculation from the initial start. Also, the fourth level can be indefinitely extended.

When meeting an acidosis it is likely more deviated and less compensated, compared to an alkalosis. Acidoses are also far more common. This means that in an acidosis it is less likely that the body can compensate and that it quickly becomes lethal itself if the underlying causes are not established and corrected. A clinical example is a respiratory acidosis in respiratory failure, where pCO_2_ levels cannot be compensated by buffering or by renal retention of bicarbonate, and the only way is to establish an adequate minute ventilation to remove pCO_2_ via the lungs [14, 16, 18–20].

Alkaloses were not as much deviated in our cohort. One explanation for this is that the normal pH range in blood is already located on the alkalemic part of the pH scale. However, alkaloses are upon presentation more auto-compensated in our cohort. This makes them also more masked with a false subnormal pH value. Because a physician meets an alkalosis more seldom, together with huge compensation and complex causes, makes them difficult to understand and to treat. One should keep in mind that the underlying problem is by no means solved although a full compensation has been reached.

In most instances the acid–base disturbance is a pure result of underlying causes and the focus must be directed towards these. In severe cases the acid–base disturbance must be corrected itself by interfering with the buffering. An acidotic state can be counteracted temporarily by administering bicarbonate (or synthetic buffers) or by increasing the minute volume in the ventilator, for instance to make the effect of vasoconstrictors more efficient, or to reverse a severe critical metabolic acidosis not compatible to life. However, tampering with the compensation gives only temporary effect and is present only in the blood compartment, and if the underlying causes are not reversed the acid–base disturbance will re-appear.

Uncommon decision pathways exist also, e.g., (1) volume contraction alkalosis by loosing acidotic fluid by vomiting, (2) hypothermia altering the speed of buffers, (3) electrolyte disturbances, such as hyperchloremic acidosis, or altered phosphate and albumin concentrations displacing the normal range of anion gap, (4) impaired cellular transporters such as in Gitelman and Bartter syndromes causing hypokalemia combined with metabolic alkalosis, (5) intoxications (salicylate stimulating the respiratory drive by direct effect on cerebral respiratory centre causing respiratory alkalosis) [1, 6, 8, 11–13].

Stewart approach has three players, pCO_2_, SID and A_TOT_, of which only pCO_2_ is directly available. We used the formula [Na^+^ + K^+^ − Cl^−^ − lactate^−^] to estimate SID; however, magnesium, phosphate, sulphate for instance also contribute to SID, but are not readily and frequently available. Our estimation of SID did not correlate well with acidity or alkalinity (12, 13). A_TOT_ is difficult to estimate and is built up by numerous contributors, such as albumin, globulins, urea, different other proteins. Therefore, a smart clinical usage of Stewart is in our opinion not easy, although there are situations, where it can in bring understanding, such as in hyperchloremic acidosis.

The script seems to work in its initial form on MATLAB. The path to a final user-experience version; either on a handheld device or as an integrated software on a device or system used in healthcare is long and includes coding the script and building a smart input module, building a user-friendly design and testing and certifying it clinically. Our next step will be to build a prototype and plan a prospective multicenter study to test it in sharp clinical situation. No script is perfect in its first version. However, we believe that this script will fast outsource manual calculation in higher versions. However, it can never work alone, it will always require that the human clinical experience is involved.

How can one ever create a complete wall-to-wall theory for the acid–base status, when it is affected by so many underlying processes? Models are created by calculating on an impaired balance between respiratory and renal functions, which are the main moderators, and further on buffers, which can be seen as temporary rescuers. Also, the accuracy of the blood gas analysator may have an impact and add up for instance in the delta ratio dependent on at least four electrolyte measurements. All available theories are simplifications and will have bugs and shadow areas, where they cannot describe what is going on. A combined understanding of acid–base theory combined with computer coding is required.

## Conclusions

Theories are developed on ideal models and are always approximations. Critically ill patients have huge disturbances in the homeostasis and are thus far from an ideal situation. All exact inputs for the models are not available in real life. On our cohort it is difficult to develop a working script based on Stewart, whereas Boston and Copenhagen work. Some instances are impossible to describe using only one theory. Blood in normal state is already alkalotic and there are more room for acidoses to deviate compared to alkaloses. The first version of the script worked well and presented a reasonable and understandable output for all blood gases calculated.

## Data Availability

Can be obtained from the corresponding author upon request..
